# Connected Skiing: Motion Quality Quantification in Alpine Skiing

**DOI:** 10.3390/s21113779

**Published:** 2021-05-29

**Authors:** Cory Snyder, Aaron Martínez, Rüdiger Jahnel, Jason Roe, Thomas Stöggl

**Affiliations:** 1 Department of Sport and Exercise Science, University of Salzburg, Schlossallee 49, 5400 Hallein/Rif, Austria; aaron.martinez@sbg.ac.at (A.M.); ruediger.jahnel@sbg.ac.at (R.J.); Thomas.stoeggl@sbg.ac.at (T.S.); 2 Athlete Performance Center, Red Bull Sports, Brunnbachweg 71, 5303 Thalgau, Austria; 3 Atomic Austria GmbH, Atomic Strasse 1, 5541 Altenmarkt, Austria; jason.roe@atomic.com

**Keywords:** IMU, principal component analysis, wearable, scoring, carving

## Abstract

Recent developments in sensing technology have made wearable computing smaller and cheaper. While many wearable technologies aim to quantify motion, there are few which aim to qualify motion. (2) To develop a wearable system to quantify motion quality during alpine skiing, IMUs were affixed to the ski boots of nineteen expert alpine skiers while they completed a set protocol of skiing styles, included carving and drifting in long, medium, and short radii. The IMU data were processed according to the previously published skiing activity recognition chain algorithms for turn segmentation, enrichment, and turn style classification Principal component models were learned on the time series variables edge angle, symmetry, radial force, and speed to identify the sources of variability in a subset of reference skiers. The remaining data were scored by comparing the PC score distributions of variables to the reference dataset. (3) The algorithm was able to differentiate between an expert and beginner skier, but not between an expert and a ski instructor, or a ski instructor and a beginner. (4) The scoring algorithm is a novel concept to quantify motion quality but is limited by the accuracy and relevance of the input data.

## 1. Introduction

Recent developments in sensor technology have made sensing units cheaper and easier to implement. These developments have made the application of “wearable technology” or smart sporting equipment appealing to not only scientists and elite athletes, but also recreational athletes. Such users are interested in more than the quantity of a movement performed (e.g., steps, ski turns, or kilometers per run); they are also interested in the quality of motion, or how well they performed the activity [1]. Together, the recent developments and new users of wearable technology have led to a number of recent publications concerning the measurement of motion quality during skiing, especially during in-field experiments [1,2,3,4].

A popular sensor choice in the field-based measurements are inertial measurement units (IMUs). These sensors combine accelerometers, gyroscopes, and optionally, magnetometers to record three-dimensional acceleration, angular velocity, and magnetic field signals. IMUs have been used to measure center of mass kinematics [5], skier posture [6], trunk orientation [7], vibration transmission [8], knee joint angles [9], edge angle [10], as well as the estimation of skier kinetics [11]. Despite the variety of approaches to quantify skiing performance, these studies focused exclusively on competitive alpine skiing [12]. Although the results of these studies provide motion quality parameters to scientists and coaches regarding athlete performance or injury risk, the methods used are not “plug-and-play” systems. In general, the methods utilized in the studies above require extensive sensor calibration, bulky measurement equipment, or offline post-processing [13]. While these processes can be quite simple, they can also be quite complex and can significantly influence data quality [14]. Indeed, this limitation was a key finding in one of the earliest publications regarding wearable systems to measure motion quality during skiing [15]. This study used an extensive sensor setup (tri-axial accelerometer, tri-axial gyroscope, force-sensing resistors, radar, and infrared distance sensors) to estimate a wide variety of parameters related to competitive skiing simulations. The main intent of this system was to provide data to augment feedback normally given by a human coach. The authors of this paper highlighted that although the measurement system was quite comprehensive, it was prohibitively obtrusive for regular everyday use, and future systems should focus on providing an interface that is easy and intuitive to operate and interpret.

More recently, there have been further developments in the area of motion quality assessment in alpine skiing. Yamagiwa and colleagues developed a simple system based on a single IMU mounted on the trunk of a skier to assess skiing quality based on turning tempo (turn frequency) [16]. The algorithm assessed only the variability of tempo during a run in order to differentiate between high- and low-skill skiers. However, this study only presented the development of the algorithm; it did not report any group statistics and included a limited number of participants. Kos and Umek [17,18] proposed a more complex system, integrating bending and load transducers directly into a ski in addition to an IMU placed on the torso of skiers. Although this system was quite comprehensive and provided real-time feedback to users, the hardware requirements (data-logger, backpack, cables) and calibration procedures (static and dynamic requirements) rendered it infeasible for realistic everyday frictionless use.

Recent work addressing the literature gap regarding low-friction systems has developed a wearable-system based on IMUs mounted on the cuffs of both ski boots and a smartphone hub for data recording, storage, and online data processing within a custom application [3]. This provides the platform for the automated detection of turns [4], data processing and extraction of skiing specific metrics [19], and turn classification into carving, drifting, or non-parallel turning styles [2]. Together, these steps fit within the activity recognition chain (ARC, segmentation, enrichment, classification) [20]. Brunauer and colleagues [21] have proposed an extension of the ARC, going beyond answering the question of “What did *X* do?”, to “How well has *X* performed?” and “What should *X* do to improve?” In order for this proposed extension to function, it requires an objective quantification of which parameters define motion quality (i.e., edge angle, radial force, CoM speed, turning radius). One approach to answer these types of questions is principal component analysis (PCA). PCA is a common tool in statistics and machine learning used to reduce the dimensionality of large time series datasets, where many variables contain redundancy with respect to the total variability of the dataset [22]. In the context of human movement analysis, PCAs have been utilized to identify unique gait patterns during walking [23], to discriminate between patients with and without knee osteoarthritis [24], and to evaluate motion quality during functional movements and classify athletes as novice or elite [25]. PCAs have also been implemented in other smart sports equipment settings—for example, to detect errors during balance board tasks [26]. In the context of skiing, PCAs have been used to identify the main motions or principal movements related to skiing technique during slalom racing [27]. While PCAs would normally be applied to an entire dataset, in the context of wearables and smart coaching, an alternative approach would be to apply a PCA model to individual time series variables in order to identify the specific components of individual parameters which contribute to overall variability. In this way, a wearable system could be developed which is more sensitive to individual parameter shapes, rather than traditional metrics such as mean, standard deviation, maximum, or minimum.

In order to develop a robust model of skiing movement quality, we develop a principal component analysis (PCA)-based model of motion quality during alpine skiing using a simple sensor system, and we evaluate the performance of the algorithm during in-field skiing conditions compared to expert raters.

## 2. Materials and Methods

### 2.1. Participants

Nineteen advanced or expert skiers (8 male/11 female, age 34.6 ± 7.8 years, height 1.73 ± 0.1 m, weight 72.7 ± 11.0 kg) were recruited to participate in this study. All participants were either ski instructors or current or former competitive alpine skiers, including four former FIS Alpine World Cup athletes. Additionally, three separate participants, one beginner, one ski instructor, and one expert skier, were recruited to complete a separate algorithm validation. Participants were informed of the testing procedures in detail, including possible benefits and risks of the investigation, prior to signing the consent form as approved by the local ethics committee (EK-GZ: 11/2018). This experiment was conducted in accordance with the Declaration of Helsinki.

### 2.2. Overall Design

In order to construct a “systematic” dataset comprising a variety of skiing styles, participants completed seven skiing runs, performing at least ten consecutive turns during each run. Participants performed carving and drifting style turns. In both styles, turns were performed in long, medium, and short radii. Long-radius turns were defined as at least three snow-cat groomed widths (>12 m), medium-radius turns were defined as roughly two snow-cat groomed widths (~8 m), and short-radius turns were defined as less than two snow-cat groomed widths (<8 m). The seventh test run was a “maximum performance” run performed at the participants self-selected turn radius and style. Additionally, participants performed one snowplow steering and one pure snowplow run; however, data from these runs were not included in the analysis. All tests were performed at three Austrian ski resorts between January and March 2019. In order to ensure consistent slope conditions, all tests were performed before 11 am. All tests were completed on freshly groomed blue or red pistes with limited fresh snowfall (<6 cm).

### 2.3. Data Acquisition

All tests were performed on commercially available recreational race skis. Long- and medium-radius turns, as well as the “maximum performance” runs, were performed on a “giant slalom” model (Atomic Redster G9 171/177/183 cm length, 18.6 m radius). Short-radius and non-parallel turns were performed on “slalom” skis (Atomic Redster S9, 155/165 cm length, 12.7 m radius). Prior to testing sessions, participants completed at least one run to familiarize themselves with the test skis.

**Figure 1:**
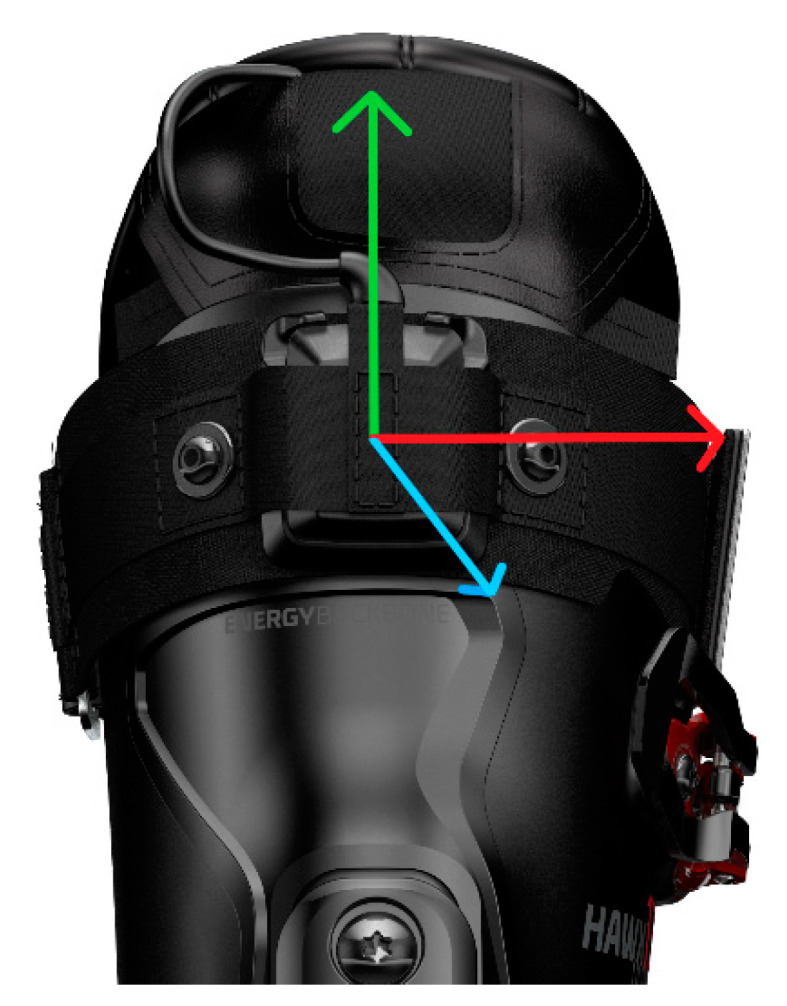
Measurement system and axis orientation. The X axis (red) points to the right, the Y axis (green) points vertically, and the Z axis (blue) points posteriorly.

The wearable system consisted of two IMUs (configuration: 2.5 × 3 × 0.83 mm ± 16 g and ±1000 dps full-scale resolution, board by Movesense [28]) mounted on the upper posterior cuff of each ski boot using a custom housing and strap. The Y axis of the IMU was aligned with the vertical axis of the boot pointing superiorly, the X axis with the lateral axis pointing to the right, and the Z axis with the roll axis pointing posteriorly (Figure 1). Both the accelerometer and gyroscope sampled at 833 Hz. The raw signals were filtered by an analog anti-aliasing low-pass filter, and again after A/D conversion by a digital low-pass filter (filter cutoff: 416.5 Hz—accelerometer; 245 Hz—gyroscope) The filtered signals were transmitted via Bluetooth at 54 Hz to a smartphone running a custom application, where they were stored for further processing. Additionally, global navigation satellite system (GNSS) signals were recorded at 1 Hz by the same custom application on the mobile phone. A central requirement of the wearable system is its “plug-and-play” character; therefore, after factory calibration, no further IMU calibrations were performed.

### 2.4. Pre-Processing

All collected data were processed according to the process outlined by the ARC proposed by Brunauer and colleagues [21]. Each run was segmented into turns using the algorithm described by Martinez et al. [3,4]. Briefly, this algorithm detects peaks in the roll axis gyroscope signal to segment turns based on the pendulum model of skiing. The first and last detected turn from each run, as well as turns with an average speed one median absolute deviation below the median speed for that run, were excluded from the dataset in order to exclude turns within each sequence where the skier was either accelerating or decelerating (i.e., the beginning and end of a run). Pre-processed data from each turn were enriched with the metrics, speed, radial force, edge angle, and edge angle symmetry (left–right turn differences) according to the algorithms proposed by Snyder and colleagues [19] and speed based on the mobile phone GNSS. Finally, each turn in the segmented, enriched dataset was classified as either carving, drifting, or non-parallel style according to the classification algorithm described by Neuwirth and colleagues [2]. Although this algorithm was able to distinguish among styles with high accuracy (~93%), not all turns within one style are similar, specifically with regard to turn size. Therefore, in order to add a further layer of specificity to the scoring algorithm, each classified turn was further classified according to the assigned turn size (small, medium, and large). Although the turn style for each run was specified, the classified turn styles assigned by the classification algorithm were not always identical to the style intended. Table 1 shows the number of turns from each participant classified in each turning style/radius, as well as the “intended” turn styles included. Due to synchronization errors, specific runs from 10 participants were excluded. These participants were all included in the test dataset, preventing their “lack” of data from influencing the model results.

The processed dataset was split participant-wise into reference (42%) and test (58%) datasets. The reference dataset was used to learn a PCA model and develop a scoring system. The test dataset was then used as “new data” to test the performance of the algorithm. The participants placed into the reference dataset were selected based on their objectively high skiing level. These five “gold-standard” skiers included one male and one female professional instructor and three retired male World Cup athletes (retired after 2006). These skiers had a combined 49 World Cup Victories, 13 World Championship medals, 4 Olympic Medals, and 9 World Cup Overall or discipline crystal globes.

### 2.5. Scoring Alogirthm

A PCA model was applied to the reference dataset to learn the signal characteristics (principal components) of the reference skiers. The principal components scores (the linear representations of each sample in the principal component space) of the first three principal components were retained and used as a reference distribution to assess the similarity between the reference and test datasets (scoring).

A centered PCA model was learned separately on each input variable (edge angle, radial force, speed, symmetry) for each skiing style in the reference dataset. Therefore, each variable is represented by an *n* × 101 dimensional matrix, *P_ref_*, where *n* is the number of samples (turns) and 101 is the number of features—in this case, the signal normalized to 100% turn duration. The PCA yields two results, a matrix of eigenvectors and a matrix of eigenvalues. The eigenvectors represent the direction of the largest sources of variability in *P_ref_* and are ordered by the magnitude of variability that they explain. These are termed principal component loading vectors ${\vec{PC}}_{ref}$. The eigenvalues of each eigenvector are the representations of the original dataset in the principal component space and are termed PC scores, $PCS_{ref},$ and they represent the amount of variability contributed by each sample to each PC loading vector. In this way, ${\vec{PC}}_{ref}$ can be thought of as a transformation matrix from the PC space and the original data space. The mean vector response of each variable and ${\vec{PC}}_{ref}$ were retained as the required data to transform new data to the PC space, where the transformed data can be scored.

While the eigenvectors ${\vec{PC}}_{ref}$ are used to transform new data into the PC space, the eigenvalues $PCS_{ref}$ are scalars which describe the contribution of each PC to the overall variability of the dataset. Therefore, assuming that the skiers in the reference dataset represent the “gold standard” of skiing performance, within one PC of one variable (ex. PC 1 of edge angle), the distribution of $PCS_{ref}$ describes the optimal weighting of that PC for that variable, where scores close to the middle of the distribution are desirable, and those at each tail are less desirable.

An absolute Z-score for each PC of each variable was calculated and transformed into discrete scoring bins, where Z < 0.75 = 4, Z > 0.75 and Z < 1.5 = 3, Z > 1.5 and Z < 3 = 2, Z > 3 = 1. These Z-scores were normalized by the variance explained by each PC and summed within variables so that PC that contributed most to the variability of the reference dataset carried the most weight in the score. The final score for each turn was calculated as the sum of normalized Z-scores across variables, expressed as a percentage of the maximum score, where higher scores represent higher similarity to the reference dataset. In order to form a single continuous scale for all turns, those turns classified as carving were scaled from 7 to 10, and those classified as drifted turns from 3to 6. Not addressed in this study were the turns classified as snowplow and snowplow steering [2]. These classes were not scored by this algorithm but assigned scores of 1 (snowplow) and 2 (snowplow steering) in order to complete the 1–10 scale. None of the turns included in this study were classified as snowplow.

The final scoring model consists of a set of vectors representing the mean response of each variable, a set of loading vector means (${\vec{PC}}_{ref}$) and loading vector standard deviations for each PC of each variable, in each turn size (small, medium, large) of each turning style (carving and drifting).

The test dataset was processed according to the same pre-processing steps as the reference dataset (segmentation, enrichment, and classification). Rather than learning a new PCA model in the incoming dataset, each variable of each metric was scaled by the mean response of the reference dataset and transformed to the PC space using the matrix ${\vec{PC}}_{ref}$. The test dataset was then scored according to the scoring algorithm described above.

Finally, three skiers completed a shortened protocol consisting of three runs in set radii (long, short, and self-selected) and self-selected turning style, using the instrumented ski boot, while being observed by three professional ski instructors. The instructors rated the skiing quality using two items, the overall quality (“On a scale of 1–4, how is the overall skiing quality? 1 being not able to ski, 4 being excellent”), the skiing dynamics (“On a scale of 1–4, how dynamic is the skiing? 1 being static, 4 being very dynamic”), and the skiing turn style (“What is the skiing style: carving, drifted, or mixed?”). The scores were scaled using the assigned style (3–6, drifting, 5–8 mixed, 7–10 carving) in order to match the scale of score provided by the wearable system. Data from the instrumented boot were processed according to the algorithm above, and the mean score from each run was compared to the scores assigned by the expert raters using Pearson correlations. Correlations less than 0.3 were interpreted as small, between 0.3 and 0.6 as medium, and greater than or equal to 0.6 as large [29]. Finally, the scores from all turns from each skier were compared using a Kruskal–Wallis test to determine if the algorithm was able to assign different scores to skiers of different skill levels.

## 3. Results

### 3.1. Explained Variability

**Figure 2:**
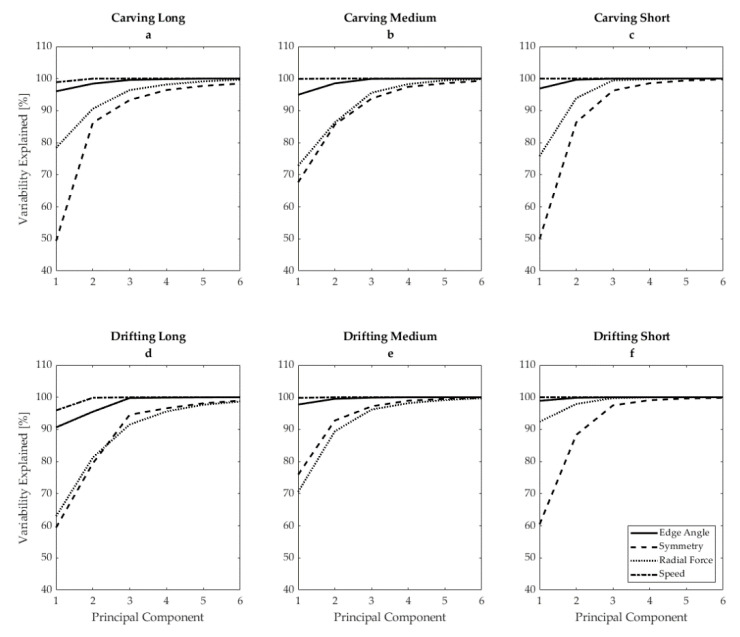
Cumulative explained variability of the first six principal components from the reference dataset for edge angle (solid line), edging symmetry (dashed line), radial force (dotted line), and speed (dot–dash line), in (**a**) carving long radius, (**b**) carving medium radius, (**c**) carving short radius, (**d**) drifting long radius, (**e**) drifting medium radius, and (**f**) drifting short radius.

The first three PCs of each variable in each turning condition explained at least 85% of the variability of the reference dataset for all variables in all skiing styles (Figure 2). The first three PCs of speed explained 99.1 ± 1.6%, 0.9 ± 1.6%, and 0.03 ± 0.1% of the total variability across all skiing styles. Similarly, the first three PCs of edge angle explained 95.9 ± 2.9%, 2.7 ± 1.4%, and 1.3 ± 1.5% of the total variability across all skiing styles. For radial force, the first three PCs explained 75.5 ± 9.9%, 4.4 ± 5.2%, and 6.6 ± 3.0% of the total variability. Finally, for symmetry, the first three PCs explained 60.4 ± 10.3%, 26.0 ± 9.1%, and 9.0 ± 3.6% of the total variability.

### 3.2. PC Response and Score Response

**Figure 3:**
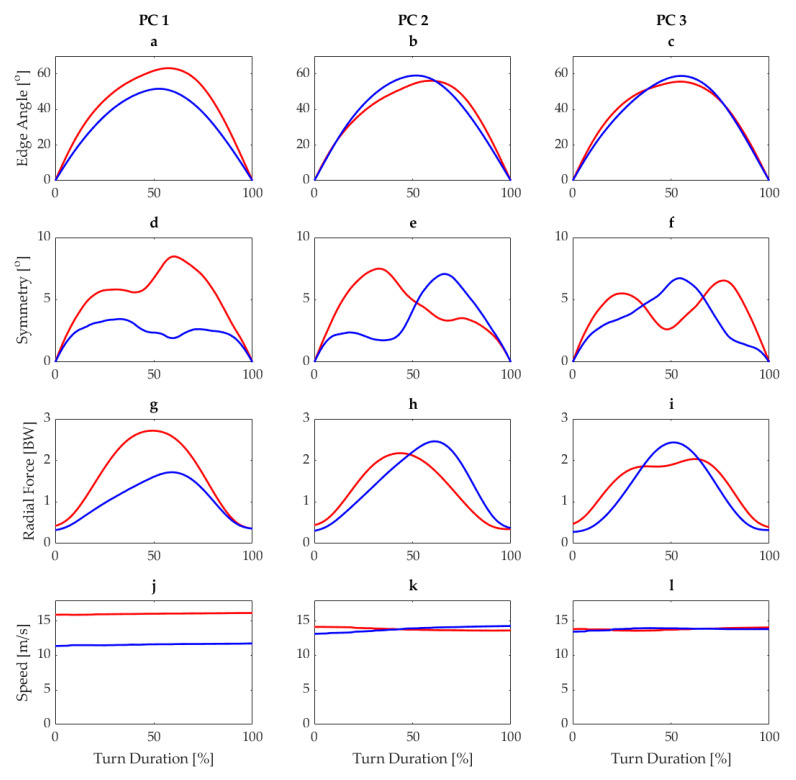
Responses for first three principal components for edge angle (**a**–**c**), edge angle symmetry (**d**–**f**), radial force (**g**–**i**), and speed (**j**–**l**) for carving short from the reference dataset. Blue lines represent the mean load score −1SD and red lines represent mean load score +1SD.

A sample response of each variable in the PC space is shown in Figure 3 for carving short radius. For example, the third row (Figure 3g–i) shows the responses of the first three PCs of radial force. Higher PC 1 scores for radial force indicate lower radial force, while lower scores indicate higher radial force. For PC 2, high scores indicate peak radial forces occurring later in turn duration, while lower PC 2 scores indicate peak radial forces earlier in the turn. Finally, in Figure 3i, higher PC 3 scores indicate single peaks in radial force, with longer transition phases where radial forces are low, while lower PC 3 scores indicate double peaks in radial force.

### 3.3. Test Score Distribution

**Figure 4:**
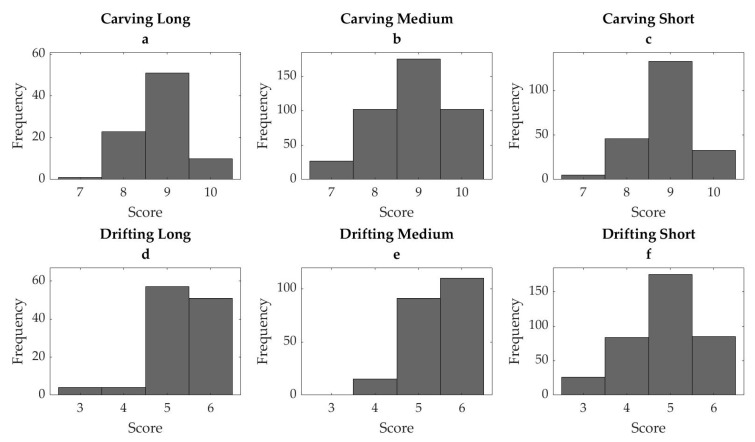
Distribution of algorithm-assigned scores from the test datasetfor the skiing styles carving and drifting in long (**a**,**d**), medium (**b**,**e**), and short (**c**,**f**) radius.

The scores assigned by the wearable system to the training dataset are shown in Figure 4. In all styles except carving medium, the scores were moderately skewed towards higher scores (carving long: −0.58, carving medium: 0.01, carving short −0.36, drifting long: −0.82, drifting medium: −0.52, drifting short: −0.38).

### 3.4. Validation Test

A large correlation was observed between the expert assigned dynamic score and the median score of all turns assigned by the wearable system within each run (r = 0.71, *p* = 0.048), but not between the overall quality score and the score assigned by the wearable system (r = 0.59, *p* = 0.120). Additionally, differences were observed between the algorithm-assigned scores for the beginner and expert skiers (*p* = 0.02), but not between the ski instructor and the expert skier (*p* = 0.23) and the beginner and the ski instructor (*p* = 0.44).

## 4. Discussion

The goal of the study was to develop a wearable sensor system-based scoring algorithm to assess motion quality during skiing. The system proposed in the current study is both well suited for use in a mobile application and is able to discriminate between high- and low-skill skiers, but only when the skiing style is sufficiently different (i.e., carving vs. drifting). Additionally, this scoring system embraces the previous steps proposed by the ARC (segmentation/enrichment/classification) and assesses motion quality relative to the performed technique and turn size [21].

A critical aspect of a scoring system is that it provides outputs which are prepared to feed further motion quality algorithms, such as the extended ARC [21]. For example, an algorithm which only provides a single numeric score is appropriate for comparing athletes or students but does not provide sufficient information to address further questions, such as “What should X do?”. As the inputs of the algorithm proposed by this paper are easily interpretable, context-relevant parameters (e.g., edge angle, radial force, speed, and symmetry), the sub-scores calculated for each feature could be used to translate the scores for each PC into concrete coaching steps.

Although a PCA is typically used to address the main sources of variability in a dataset and reduce the number of input variables, in this case, PCAs are applied to each signal separately in order to identify the main variability sources within each signal independently, specifically so that the results could be translated into context-relevant coaching instructions. For example, consider a skier whose lowest sub-score comes from the radial force variable. Their PC 1 score was lower than the target. A low PC 1 score for radial force indicates that they ski with higher radial force than the target so they should aim for a lower radial force turn. Their PC 2 score was higher than the target, which, for radial force, indicates that the radial force peak was later in the turn. Therefore, they should aim to have their peak radial force earlier in the turn. Finally, for PC 3, their score was similar to the target, so the duration of the turn with higher radial force was similar to the reference.

Additionally, the scoring algorithm incorporates signals related to multiple aspects of alpine skiing and is able to assess the motion quality across all of the aspects independently of each other. For example, although skier A achieved a good score for PC 1 of edge angle, they received a low PC 2 score: both of these aspects are related to skiing performance; however, the magnitude of edge angle is more important for the timing of the peak edge angle, as, during carving skiing, the edge angle is directly related to the turn radius [30]. This is also reflected in the PCA results, as the variance explained by PC 1 (related to signal magnitude) in all skiing styles and all variables explained 82 ± 17% of the total variance, while PC 2 and PC 3 (generally related to signal timing and duration) explained 14 ± 15% and 3 ± 4% of the total variance of the dataset, respectively. The algorithm also considers this fact, scaling the contribution of each PC to the total score for each variable by the variance explained for each PC. For example, if a skier were to score a perfect 4 in each PC of radial force, their scores would be 2.78, 0.88, and 0.34, as PC 1–3 explain 70%, 22%, and 5%, respectively, of the total variance in the radial force signal. This prevents poor scores in PCs, which contribute very little to the overall variability from exacting an outsized influence on the overall score—for example, speed, where PC 1 explains ~99% of the variability across all styles. In the case of speed, the 1 Hz GPS signal is linearly interpolated to the 54 Hz IMU sample frequency by the application. Therefore, only the magnitude of the signal and not the shape of the signal is meaningful. In this case, the PCA is also “smart” enough to treat this parameter in the same way that a maximum value only would be scored.

The primary consideration for a scoring algorithm is its ability to distinguish between high- and low-skill skiers. In general, it can be observed that higher scores are associated with higher edge angles, higher symmetry, higher radial force, and higher speed, which generally matches the assumptions of higher performance observed in competitive environments [12]. The simplest proof of concept would be to use the algorithm to compare two skiers, one retired WC athlete and one beginner skier. The beginner skier was able to complete drifting turns with a low motion quality, but not carving turns. Given that the two skiers in this test completed the same test protocol, the mean score from the two skiers should represent their overall skiing quality. The average score for skier A, the retired WC athlete, across all collected turns was ~8.4, reflecting their high motion quality even in “lower-skill” drifted turns. The average score for skier B, the beginner, was 5.9. Although this skier was instructed to perform carving turns, according to the algorithm, this skier was unable to perform carving turns, and thus a majority of their turns were classified as drifting and thus scored below 7.

**Figure 5:**
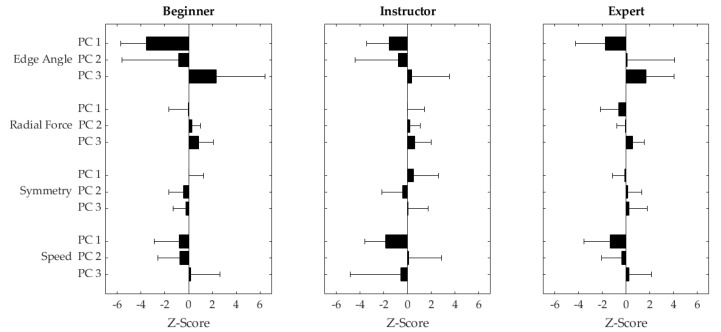
Mean +/− 1 standard deviation Z-transformed PC scores for PC 1–3 (top to bottom) across all runs (long, short, self-selected) for a beginner skier (**left**), a ski instructor (**middle**), and an expert skier (**right**).

The results of the in-field validation show that the scoring algorithm was correlated with scores representing skiing dynamics, but not with the overall skiing quality. Therefore, it appears that the algorithm scores movement quality more based on the dynamics of the movement than the subjective motion quality as assessed by expert raters. This is a logical outcome, since the dynamics were the parameters directly measured by the IMU system (acceleration and angular velocities). Although the algorithm was able to correctly rank the skills of the three test skiers (beginner < instructor < expert), and the scores assigned accurately distinguished between the beginner and expert skier, Figure 5 shows that, outside of edge angle and speed, the scores were generally quite similar across all variables. Due to these similarities, the scores assigned by this algorithm are likely only discriminant enough to differentiate between skiers when the skiing style is sufficiently different (higher vs. lower dynamics).

The observation that the algorithm scores skiers based on their skiing dynamics highlights the importance of the choice of skiers included in the reference dataset. The scores assigned by the algorithm to the test dataset were generally skewed towards higher scores, which would be expected since the skiers in this dataset were all either instructors or former competitive alpine skiers. Although the reference group in this study contained only elite alpine skiers (retired WC athletes), the algorithm only assesses how similar the variability new data was to the set of reference skiers for each individual skiing style. Therefore, it is possible to receive a negative score when PC scores are either above or below the reference skiers’ values. For example, a skier with extremely high edge angles might receive the same score as a skier with a lower edge angle because they are both equally different from the reference dataset but in opposite directions, even though the case of the extremely high edge angle indicates objectively better skiing quality. Additionally, it is possible that the variability contained in this dataset (containing only expert skiers) might not represent the variability that might be contained by a dataset that includes more intermediate or novice skiers. On the contrary, such a dataset might not generate meaningful targets, as the variability of such a dataset would represent the variability between skill groups rather than the variability of a reference group. This highlights the importance of the selection of an appropriate reference group, which should match the quality of skiing to be evaluated by the algorithm.

In this context, a distinct advantage of this algorithm is its flexibility in adapting to a new reference. A user could simply record a new selected group of reference skiers, and the algorithm could learn a new reference model based on this new dataset, provided that it contained an appropriate volume of turns in all desired skiing styles. This could also be done on an individual level to provide a baseline for competitive skiers to detect subtle changes in skiing technique possibly related to fatigue and increased injury risk during longer training sessions [31].

A limitation of an approach such as the extended ARC is the issue of dependency. The accuracy of each step in the extended ARC is dependent on the accuracy of the previous step. For example, the accuracy of the ski style classification is dependent on the accuracy of the feature extraction step, which is dependent on the accuracy of the segmentation step. Despite this limitation, all of the steps in the extended ARC proposed above have been previously validated. Martinez and colleagues performed in-lab [3] and in-field [4] validations of the turn detection and segmentation algorithm, and the accuracy and precision of the estimated features edge angle and radial force have been shown to be −0.77 ± 1.00° and 1.50 ± 1.33°, respectively, and the classification algorithm proposed by Neuwirth and colleagues was able to distinguish between drifting and carving turns with an accuracy of 95%. This indicates that the error contributed from previous processing steps likely imposes a minimal influence on the accuracy of the scoring system. Additionally, the algorithm was designed based on input data processed via the same input algorithms, and, thus, the error contributed by the system itself is inherently included in the model of skiing motion quality.

## 5. Conclusions

The scoring algorithm presented in this study is a first step towards developing a wearable system to evaluate skiing motion quality that could be implemented in a stand-alone application for regular use by both recreational and competitive alpine skiers. The proposed system is a novel approach to quantifying motion quality and was able to differentiate between high- and low-skill skier abilities. Additionally, the system is easy to use, flexible, and could be easily adapted to accommodate reference skiers of varying abilities. Future work should focus on validating the algorithm in a wider range of skiing conditions, such as powder or moguls, using a more accurate reference scoring and incorporating further features (e.g., pressure distribution, ski bending, or mechanical energy dissipation) into the algorithm in order to provide a more robust and comprehensive view of motion quality during alpine skiing [12].

## Figures and Tables

**Table 1:** Turns classified per participant as carving or drifting in long, medium, and short radii. Although participants were instructed to complete turns of specific styles, some turns were classified as different styles or radii. Experience levels: WC corresponds to retired world cup ski racers, while FIS corresponds to retired FIS level ski racers.

#	Experience Level	Group	*Carving*			*Drifting*			Completed Runs
			Long	Medium	Short	Long	Medium	Short	
1	WC	Ref	10	42	29	9	25	1	CL, CM, CS, DL, DM, DS, Max
3	WC	Test	3	4	0	~	~	~	CL
4	Instructor	Test	~	~	~	~	~	34	DS
5	Instructor	Test	~	~	~	~	~	37	DS
6	FIS	Test	~	~	~	~	18	10	DS
7	Instructor	Test	~	~	~	~	1	23	DS
8	Instructor	Test	~	~	12	~	~	22	CS, DS
9	Instructor	Test	~	~	5	~	2	18	DS
11	Instructor	Test	~	~	~	~	2	15	DS
12	Instructor	Test	1	10	7	~	~	17	CM, DS, Max
14	Instructor	Test	15	16	2	8	11	2	CL, CM, CS, DL, DM, DS, Max
15	FIS	Test	4	42	22	5	17	47	CL, CM, CS, DL, DM, DS, Max
16	Instructor	Test	~	33	4	7	43	35	CL, CM, CS, DL, DM, DS, Max
17	Instructor	Ref	2	73	12	14	24	3	CL, CM, CS, DL, DM, DS, Max
19	Instructor	Ref	8	18	30	6	12	5	CL, CM, CS, DM, DS
20	Instructor	Test	5	54	25	12	22	45	CL, CM, CS, DL, DM, DS, Max
21	FIS	Test	13	21	14	6	16	20	CL, CM, CS, DL, DM, DS, Max
23	WC	Ref	10	59	59	20	6	27	CL, CM, CS, DL, DM, DS, Max
24	WC	Ref	11	43	~	21	9	~	CL, CM, DL, DM, Max
Total			82	415	221	108	208	361	84

## Data Availability

The data presented in this study are available on request from the corresponding author.
